# Urban memory narratives on social media, place attachment among internal migrants, and life satisfaction: a case study of Dongguan

**DOI:** 10.3389/fpsyg.2026.1887411

**Published:** 2026-07-21

**Authors:** Yunfeng Ye, Huifang Liu, Wenhui Kuang, Wenxuan Chen

**Affiliations:** 1 Brand Center, Dongguan City University, Dongguan, China; 2 School of Marxism, Dongguan City University, Dongguan, China; 3 School of Language and Culture, Dongguan City University, Dongguan, China

**Keywords:** Dongguan, life satisfaction, migrants, place attachment, social media, urban memory

## Abstract

**Introduction:**

As social media becomes embedded in urban life, digital urban memory narratives serve as an important channel through which internal migrants encounter local culture and life. This study examines the statistical associations among perceived social media urban memory narratives, narrative transportation, place attachment, urban identity, and life satisfaction in Dongguan, China. In this study, these narratives refer to story-based digital content connecting urban history and labor experiences with migrants' everyday lives, rather than general city branding or news exposure, or promotional city-image communication.

**Methods:**

An online survey yielded 890 valid responses from internal migrants. Data were analyzed using hierarchical regression, confirmatory factor analysis (CFA), Bootstrap mediation (5,000 resamples), and multi-group comparison based on length of residence.

**Results:**

Core scales demonstrated acceptable internal consistency (α = 0.813–0.906; CR = 0.870–0.920). The seven-factor measurement model showed an acceptable fit, χ^2^/*df* = 2.85, CFI = 0.892, RMSEA = 0.068. Perceived urban memory narratives and narrative transportation were positively associated with place attachment. Both place attachment and urban identity were positively associated with life satisfaction. Furthermore, place attachment mediated the association between perceived narratives and life satisfaction, explaining 29.6% of the total association. Length of residence, however, did not moderate relationships.

**Discussion:**

These findings highlight that story-based digital narratives provide a vital symbolic and emotional context for understanding migrants' psychological and spatial integration. Future longitudinal research is needed to clarify the temporal ordering and causal pathways of these digital emotional processes.

## 1 Introduction

Urban memory was originally more closely associated with memorial buildings, local chronicles, museums, old neighborhoods, and community narratives. As media forms such as short videos, WeChat official accounts, video accounts, Xiaohongshu, and local life platforms continue to enter urban life, urban memory has begun to be re-presented in lighter, more fragmented, and more everyday ways. The China Internet Network Information Center (CNNIC) (2024) indicates that social media and short video platforms have become important channels for the public to obtain information, participate in public life, and access cultural content. In this context, urban memory is more likely to enter the public's information flow in an image-based, story-based, and interactive manner (Li and Alencar, 2023; Liu and Zhang, 2024). Stories of old factory renovations, historical images of Keyuan and Humen, life fragments of Guancheng old streets, struggle narratives of migrant workers, and personal memories of urban village renewal projects may all enter the information flow of migrants through social media. Thus, the city is no longer just a geographical space to be viewed, but also a socio-psychological field to be narrated, commented on, forwarded, and re-understood.

In this study, social media urban memory narratives are understood as a specific type of place-based narrative content. They do not simply refer to all Dongguan-related online information, nor are they equivalent to city branding, local news exposure, or official city-image communication. Rather, they refer to digital narratives that connect recognizable urban spaces with remembered histories, cultural symbols, labor experiences, neighborhood life, and ordinary people's stories. Their distinctiveness lies in the combination of three features: memory orientation, narrative form, and experiential proximity. That is, such content presents the city through remembered or reinterpreted pasts, organizes information through stories and scenes, and links urban change with everyday life experiences that migrants may recognize or compare with their own.

Dongguan provides a relatively representative research context for this issue. As an important manufacturing city in the Pearl River Delta, Dongguan has long had a large-scale inflow population. In the context of this study, the term “internal migrants” refers to individuals who have moved to Dongguan from other regions within China for work and life, encompassing both rural-to-urban laborers and cross-city professionals. These individuals are sometimes characterized as “new citizens” in Chinese urban governance discourse, but this paper uses “internal migrants” as the main analytical term in order to maintain consistency with migration studies and to avoid conflating governance terminology with the broader migrant population examined here. For many, the starting point for coming to Dongguan is job opportunities, not cultural identity; their entry into the city is usually through factories, rental houses, commuting routes, commercial districts, communities, and acquaintance networks. For this group, place attachment does not necessarily grow out of a grand urban image, but often comes from specific life details: a familiar intersection, a repeatedly passed road, a video telling the story of an ordinary worker, or a short film about the city's changes. Consequently, a question worth asking emerges: When urban memory is re-narrated through social media, is there an observable statistical relationship between it and migrants' place attachment, urban identity, and life satisfaction?

In existing studies, the new media presentation of urban memory is often discussed within the framework of city branding, city image, or cultural communication. Such studies help explain how cities are packaged, communicated, and viewed, but rarely touch further on the psychological experience of the audience perspective, especially how migrants understand the city, settle their own experiences, and form local emotions in social media narratives. This leaves room for a more migrant-centered perspective: instead of asking only how a city communicates its image, it is also necessary to ask how migrants encounter, interpret, and emotionally locate themselves within digital stories about the city. At the same time, place attachment research emphasizes the emotional connection and functional dependence between individuals and places, while urban identity concerns whether individuals understand themselves as members of the urban community. These two constructs are related but not identical: place attachment emphasizes the person–place bond, whereas urban identity emphasizes the person–community or person–group relationship. In addition, subjective wellbeing research focuses on individuals' overall evaluation of their life status, but the present study measures this outcome primarily through life satisfaction, which represents the cognitive evaluation dimension of subjective wellbeing rather than the full range of affective and psychological wellbeing. Combining these perspectives under the framework of digital placemaking reframes “urban memory” not merely as communication content, but as an active digital process through which migrants negotiate place attachment and urban identity, thereby serving as an empirical entry point for observing the psychological integration of migrants.

For Dongguan, this issue also has clear practical implications. As a manufacturing and population-inflow city, Dongguan's urban memory exists not only in historical buildings and cultural landmarks, but also in the intersecting spaces of factories, rental houses, urban villages, commuting roads, industrial parks, and old and new urban areas. For a large number of migrants, their relationship with Dongguan is often gradually formed through work, residence, socializing, and media exposure. Urban memory narratives on social media may not directly change individuals' living conditions, but may provide a supplementary path for them to understand the city, identify their positions, and establish local emotions. Such narratives may be especially meaningful because they place migrants' everyday experiences within a broader urban landscape, transforming mediated local scenes into symbolic resources through which migrants interpret their own urban lives.

This study adopts a cross-sectional questionnaire survey method to address the following questions: What are the patterns and levels of Dongguan migrants' exposure to urban memory content on social media? What is the correlational structure among perceived urban memory narratives, narrative transportation, place attachment, urban identity, and life satisfaction? Does place attachment present a mediating association between perceived urban memory narratives and life satisfaction? Does the length of residence constitute a noteworthy boundary condition? Here, narrative transportation refers to the degree to which individuals become cognitively and emotionally absorbed in story-based content, serving as a key media-related psychological experience associated with place attachment.

It should be noted that this study does not attempt to explain social media narratives as a single, direct source of psychological variables. A more cautious understanding is that social media provides a supplementary path for migrants to contact urban history, daily life, and local culture, and this path intertwines with their actual residential experience, social relationships, and identity understanding in the city to jointly construct an investigable and describable psychological experience. Statistical results based on cross-sectional data should also be understood as association structures between variables, rather than strict temporal sequences or causal chains.

## 2 Literature review and research hypotheses

### 2.1 Urban memory narratives in social media

Urban memory includes not only tangible resources such as historical buildings, industrial heritage, and cultural landmarks, but also everyday experiences such as labor experiences, migration stories, neighborhood life, and local customs. Urban memory is not just the static preservation of urban historical facts, but also a form of collective memory continuously constructed and reinterpreted within social groups, spatial experiences, and everyday narratives (Halbwachs, 1992). Old neighborhoods, industrial heritage, cultural landmarks, and everyday urban spaces can all become “lieux de mémoire” (sites of memory) that carry urban memory, and are re-narrated and reproduced in the new media environment (Nora, 1989). The rise of social media and short video platforms has changed this communication landscape, allowing local culture, city news, intangible cultural heritage content, and ordinary people's life stories to be disseminated in more visualized, contextualized, and story-based ways (Liu and Zhang, 2024; Zort et al., 2023). Digital placemaking research also suggests that migrants and city dwellers can re-understand, connect with, and participate in the construction of place meaning through digital media (Li and Alencar, 2023; Zhao, 2025). In this study, social media urban memory narratives refer specifically to story-based digital content that links urban history, cultural memory, spatial change, and ordinary life experiences; they are therefore narrower than general local news exposure, city-brand communication, or promotional city-image content.

In the context of Dongguan, urban memory has a unique structure. It contains traditional cultural resources such as Lingnan culture, Keyuan, Humen history, and intangible cultural customs, as well as modern experiences such as reform and opening up, manufacturing development, the lives of migrant workers, and urban renewal. For migrants, this content does not merely serve as city image material, but may also intersect with their own experiences. For example, a video about factory changes may remind respondents of their own employment experiences; a set of old street photos may evoke their re-understanding of the rhythm of urban life. Thus, urban memory narratives are not just about “what Dongguan is,” but also about “how I understand my relationship with Dongguan.”

Urban memory narratives on social media also have obvious participatory features. Audiences can like, comment on, share, or add their own life experiences in the comment section. For migrants, this participation does not necessarily appear as formal public expression, but more often as a lightweight emotional confirmation: seeing a video of a familiar street, reading a worker's story, or finding a similar experience in the comments. Urban memory thereby transforms from static historical materials into media content that can be daily accessed, instantly responded to, and personally understood.

Based on the above discussion, this study focuses on the perceived exposure to urban memory narratives on social media among migrants and examines its statistical relationship with place attachment. If social media urban memory content can evoke respondents' attention to Dongguan's history, culture, space, and ordinary people's life stories, it may form a certain association with their sense of closeness, meaning, and functional dependence on the city.

Therefore, the following hypothesis is proposed:

H1: Perceived social media urban memory narratives are significantly positively correlated with migrants' place attachment.

### 2.2 Narrative transportation and media psychological experience

Narrative transportation theory focuses on an individual's attentional focus, imaginative participation, emotional involvement, and sense of entering a situation when exposed to narrative content. Green and Brock (2000) introduced narrative transportation theory, emphasizing that when an audience psychologically enters the story world, their cognitive, emotional, and imaginative activities are drawn in by the narrative content. Subsequent studies further developed brief measurement tools for narrative transportation, providing an operational basis for communication and media psychology research (Appel et al., 2015). Unlike simple information reception, narrative transportation emphasizes the audience's psychological participation in story scenes and character experiences. When social media content is organized around people, places, changes, and emotions, the audience is more likely to form specific mental images. Related research also shows that the vividness and sense of presence of media content affect audiences' psychological involvement and emotional responses (Chen and Yao, 2022; Zhu et al., 2025).

Urban memory narratives inherently possess spatial context. Old neighborhoods, factories, landmarks, markets, communities, parks, and commuting routes can all become the spatial anchors of a narrative. For migrants, if these scenes are close to their personal life experiences, they may more readily develop an immersive experience. For example, a short video about the transformation of an old industrial area might make a respondent connect urban changes with their own work experiences; an article with pictures about the everyday atmosphere of an urban village might evoke a sense of familiarity with their daily living environment. At this point, the audience is not just receiving information, but is entering the urban narrative situation through imagination, emotion, and experiential recall.

There may be a close statistical relationship between narrative transportation and place attachment. The more an individual can form attentional focus, emotional resonance, and situational imagination in urban memory content, the more likely they are to understand urban space as a place related to their own life. Narrative transportation is therefore treated here as a media-related psychological experience that helps describe how migrants engage with urban memory content, rather than as a confirmed causal mechanism. It should be emphasized that this study does not understand narrative transportation as the unidirectional source of place attachment, but examines whether a stable association is presented between the two within the scope of cross-sectional data.

Therefore, the following hypothesis is proposed:

H2: Narrative transportation is significantly and positively correlated with migrants' place attachment.

### 2.3 Place attachment, life satisfaction, and mediating associations

Place attachment is generally understood as the emotional connection and functional dependence formed between individuals and specific places. Williams and Vaske (2003) distinguished place attachment into two classic dimensions: place identity and place dependence. The former emphasizes the relationship between the place and the self-concept, while the latter emphasizes the functional value of a place for living, working, recreation, or development. Although place attachment includes a place identity dimension, this study uses it primarily to capture migrants' emotional and functional bond with Dongguan as a place.

Migrants' place attachment is procedural. Many people come to Dongguan not because of pre-existing cultural affinity, but because they are attracted by job opportunities, family migration, social networks, or economic conditions. Place attachment does not exist from the beginning; it may gradually form over long-term residence, job stability, social support, contact with urban culture, and the accumulation of personal experiences. For migrants, “whether they like Dongguan” is not an abstract question, but is often related to whether they have found job opportunities, established a social circle, formed familiar routes, and possess dependable relationships here. In migrant studies, place attachment is also seen as an important psychological variable for understanding urban integration, settlement intention, and sense of belonging. Existing studies have found that migrants' perception of the city, social relationships, and spatial experiences are associated with their city attachment and settlement intentions (Hu et al., 2024; Lu et al., 2024; Xie and Huang, 2022). At the same time, the digital economy and digital media environment may further affect migrants' urban integration process by changing the ways they access information, participate socially, and connect with the city (Zou and Deng, 2022).

Subjective wellbeing is a broad construct, while this study focuses specifically on life satisfaction as its cognitive evaluation dimension. Life satisfaction is an important cognitive evaluation dimension in subjective wellbeing research, and the Satisfaction With Life Scale is one of the widely applied classic measurement tools (Diener et al., 1985). Considering the actual measurement content of the questionnaire in this study, this paper mainly understands and measures subjective wellbeing from the cognitive evaluation dimension of life satisfaction, i.e., respondents' overall satisfaction with their current living status in Dongguan. For migrants, subjective wellbeing is not only related to income, housing conditions, and job stability, but also to urban adaptation, social support, and local emotions. Whether a person feels they “have a place here,” and whether they can establish familiar life networks in an unfamiliar city, often enters their life satisfaction evaluation. Existing studies show that there is an association between social media exposure and individual subjective wellbeing, but this relationship may be affected by usage patterns, social relationships, and individual differences (Yang and Feng, 2024). In addition, place attachment has also been found to be associated with residents' life satisfaction, suggesting that the emotional and functional connection between individuals and places may enter their evaluation of life quality (Pai et al., 2024).

In a migrant-receiving city such as Dongguan, life satisfaction comes not only from urban resources themselves but also from whether individuals can transform these resources into perceivable living security, sense of belonging, and development possibilities. If social media urban memory narratives connect with individual experiences, they may also relate to life satisfaction through the psychological mechanism of place attachment. Place attachment is selected as the mediating construct because it directly captures migrants' emotional and functional relationship with the city, which is theoretically close to the spatial and experiential nature of urban memory narratives. In other words, urban memory content does not simply correspond directly to life satisfaction, but may enter individuals' overall evaluation of their life status through the intermediate link of “my relationship with this city.” Based on the cross-sectional survey, this study formulates it as a mediating association without making strict causal inferences.

Therefore, the following hypotheses are proposed:

H3: Place attachment is significantly positively correlated with migrants' life satisfaction.

H4: Place attachment presents a mediating association between perceived social media urban memory narratives and life satisfaction.

### 2.4 Urban identity and psychological integration

Urban identity is related to place attachment, but they represent distinct psychological structures. While place attachment captures the emotional and functional bond between individuals and a specific location (i.e., “how close I feel to Dongguan”), urban identity emphasizes social categorization and membership in the urban community (i.e., “whether I see myself as a member of this city”). In other words, place attachment leans toward the geographical-functional link between the individual and the physical environment, whereas urban identity represents the ideological identification with the host community. This distinction helps minimize conceptual overlap, framing them as related but independent dimensions of migrants' psychological integration.

For migrants, urban identity is an important manifestation of psychological integration. If migrants are willing to introduce Dongguan to others or feel happy when Dongguan is evaluated positively, it indicates that their urban life experience has partially entered their identity structure. Place attachment and urban identity together constitute two important aspects of migrants' psychological integration. Place attachment may be more closely related to emotional closeness and functional dependence, while urban identity may be more closely related to membership and recognition. Although these two dimensions are naturally expected to correlate positively as concurrent facets of local integration, urban identity is further expected to play an independent, direct role in shaping migrants' overall cognitive evaluation of their lives by fostering social connectedness and reducing alienation.

Therefore, the following hypotheses are proposed:

H5b: Urban identity is significantly positively associated with migrants' life satisfaction.

### 2.5 Boundary conditions of length of residence

Length of residence is an important but complex variable in migrant studies. Generally speaking, the longer the residence time, the more likely individuals are to accumulate more local life experiences, social relationships, and spatial familiarity. However, urban experiences in the digital media environment do not completely depend on offline time accumulation. From the perspectives of cognitive schema formation and information integration theory, individuals continuously synthesize media-delivered symbolic representations with direct physical encounters to evaluate and connect with their environments. Social media allows urban history, local culture, and ordinary people's stories to enter the information flow of groups at different residence stages with a lower threshold. Existing studies show that residence experience, social relationships, urban integration, and the digital environment may all be associated with migrants' place attachment, urban belonging, and settlement intentions (Hu et al., 2024; Lu et al., 2024; Xie and Huang, 2022; Zou and Deng, 2022).

For migrants who have not been in Dongguan for long, urban memory content on social media may become an entry point for understanding the city. When offline experiences have not yet fully unfolded, short videos, graphic pushes, and local account content serve as vital external cues to facilitate their initial cognitive schema formation, helping them form a preliminary understanding of the city. For those who have lived there longer, they have already accumulated many urban experiences through work, family, communities, and life routes. According to information integration theory, for these long-term residents, urban memory content on social media may also reactivate their past experiences and strengthen their understanding of urban changes and personal life trajectories.

Therefore, whether the length of residence will change the statistical relationship between perceived urban memory narratives and place attachment cannot be simply presupposed. Compared with longer-residing migrants, newer migrants may rely more on social media urban memory narratives to develop initial familiarity with the city; therefore, the association between perceived urban memory narratives and place attachment may be stronger among migrants with shorter residence duration. Based on theoretical boundary conditions, this study incorporates length of residence into a moderating association test.

Therefore, the following hypothesis is proposed:

H6: The positive association between perceived social media urban memory narratives and place attachment is stronger among migrants with shorter residence duration than among those with longer residence duration.

**Figure 1:**
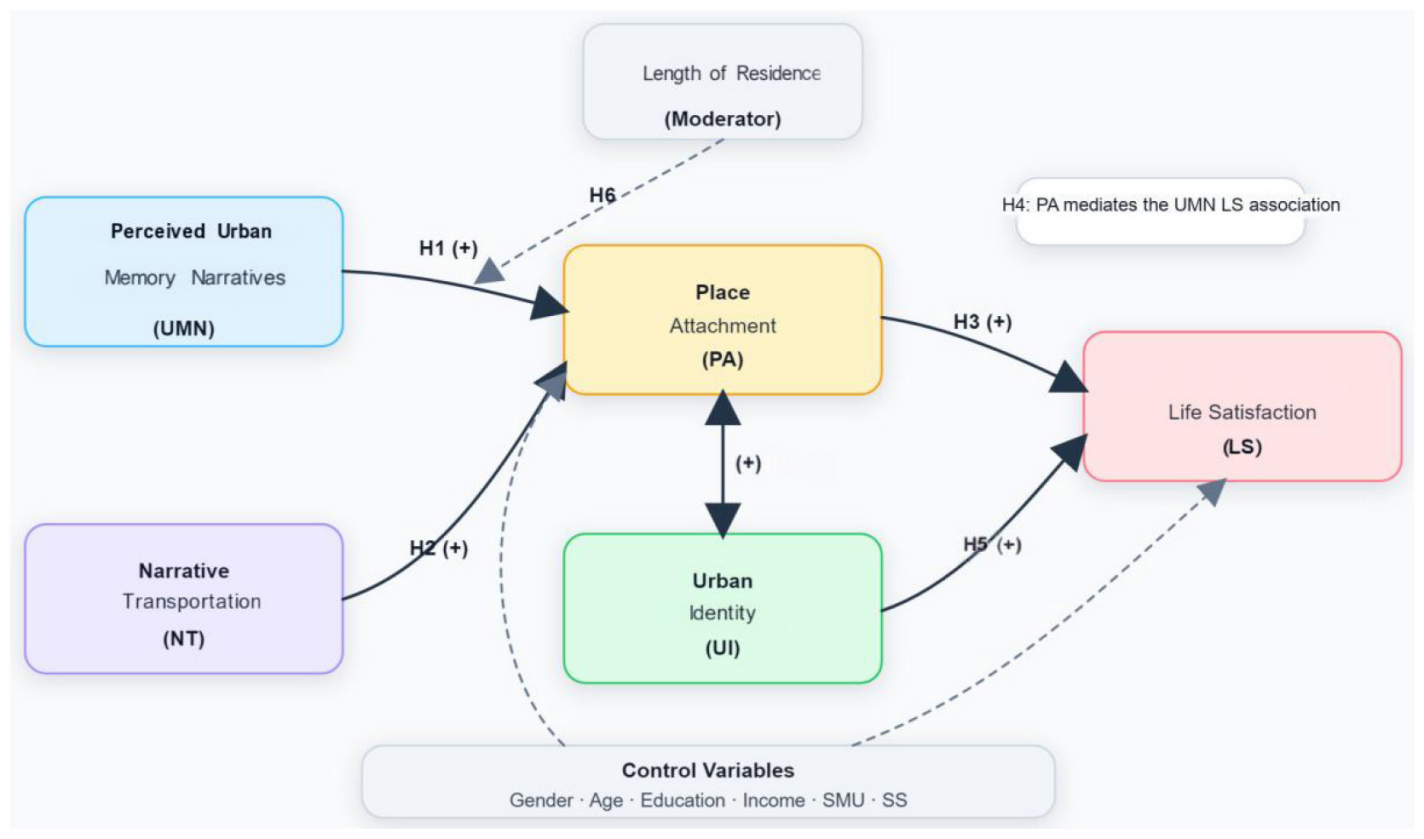
Research theoretical model and hypothesis paths. UMN, Perceived social media urban memory narratives; NT, Narrative transportation; PA, Place attachment; UI, Urban identity; LS, Life satisfaction; SMU, Social media use integration; SS, Social support. Single-headed solid arrows represent hypothesized positive associations. The double-headed arrow between PA and UI indicates a correlation rather than a causal direction. Dashed arrows indicate the moderating role of length of residence and the inclusion of control variables in the regression and mediation models.

Based on the above theoretical discussion, this paper constructs a theoretical research model to present the hypothetical relationships, mediating mechanisms, and moderating conditions among core variables, specifically as shown in Figure 1.

## 3 Research design

### 3.1 Sample and data collection

**Figure 2:**
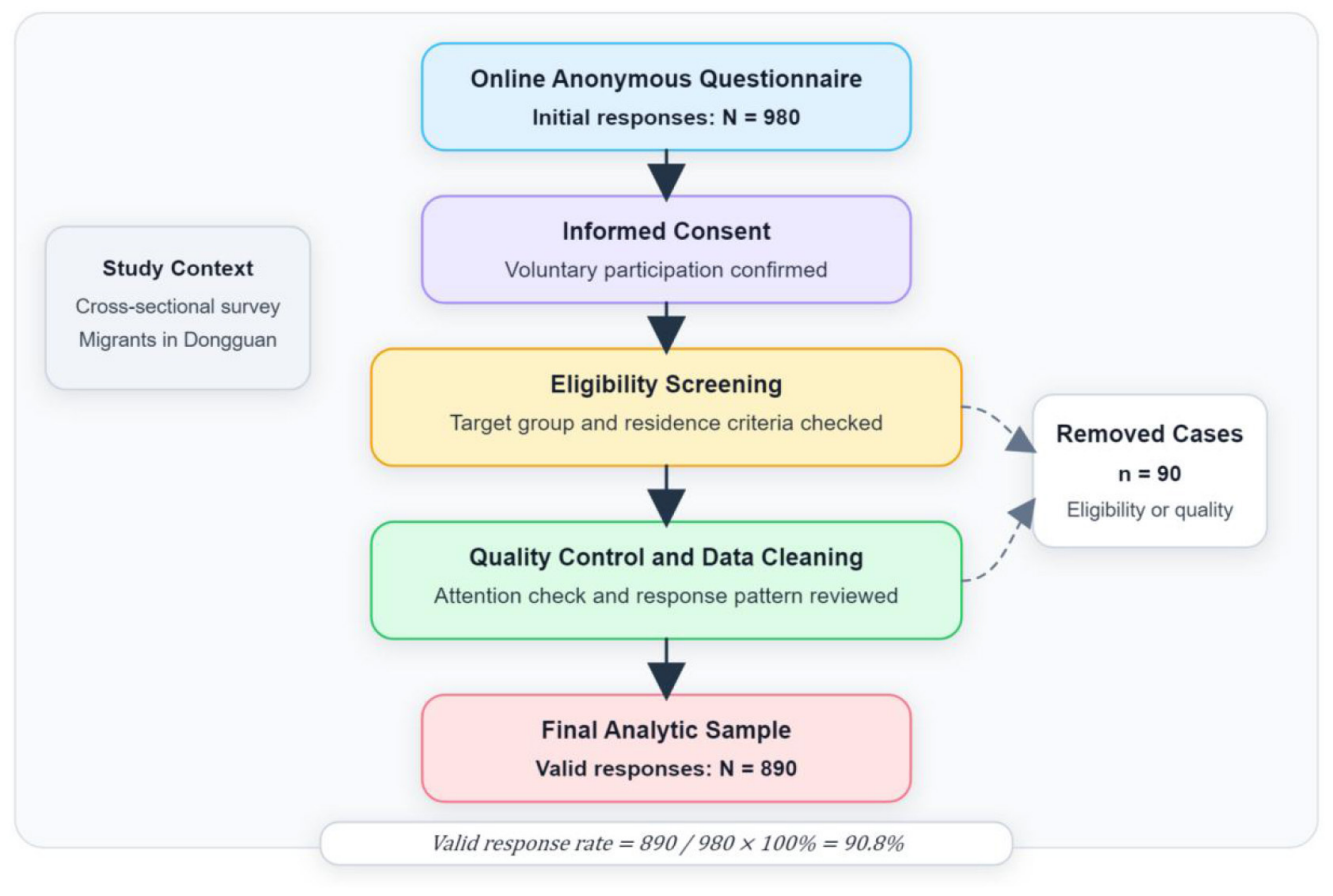
Sample screening and data cleaning process. This study conducted an online anonymous questionnaire survey through the Wenjuanxing platform. Inclusion criteria included: aged 18 or above, currently living, working, or studying in Dongguan, cumulative residence length exceeding 6 months, and not born or raised for a long period in Dongguan. Excluded samples included questionnaires that did not meet the inclusion criteria, failed attention checks, or showed obvious regular response patterns. The valid response rate is calculated by dividing the final number of valid samples by the initial number of collected questionnaires, 890/980 × 100% = 90.8%.

This study targets migrants who live, work, or study in Dongguan, to explore the statistical associations between social media urban memory narratives, place attachment, and life satisfaction. Data collection was conducted online anonymously via the “Wenjuanxing” platform from February 15 to April 15, 2026. The questionnaire link was distributed through Dongguan-related social media groups, community networks, workplace and industrial-park networks, and local life-information platforms, so as to reach migrants with different occupations, residence areas, and social media use patterns. No quota sampling was used, resulting in a non-probability convenience sample. This study set strict sample inclusion criteria: (1) Adults aged 18 and above; (2) Not born or raised for a long period in Dongguan; (3) Currently living in Dongguan with a cumulative duration of more than 6 months. All respondents had to read an informed consent form and voluntarily click to confirm before entering the formal questionnaire. This survey collected a total of 980 questionnaires. After applying the inclusion criteria, attention checks, and low-quality response identification, 890 valid samples were finally obtained, with a valid response rate of 90.8%. The multi-channel distribution strategy helped reduce the risk of relying on a single platform or single occupational group. To enhance the transparency of the sample screening process, this paper visualizes the questionnaire recovery, inclusion criteria screening, quality control, and final valid sample formation process, specifically as shown in Figure 2.

### 3.2 Research instruments

The questionnaire includes modules on screening questions, demographic variables, social media use, social media use integration, perceived urban memory narratives, narrative transportation, place attachment, urban identity, life satisfaction, and social support. Among them, life satisfaction is used as the cognitive evaluation dimension of subjective wellbeing in this paper.

Demographic and background variables include gender, age, education level, marital status, occupational identity, household registration status, living arrangement, monthly income, length of residence, and intention to stay in Dongguan long-term. Social media use includes frequency of use, daily usage time, commonly used platforms, whether they have contacted Dongguan urban memory-related content in the past 3 months, forms of contact, and preferred types of urban memory content. Multiple-choice questions were coded using a multiple-dichotomy method, where each option was split into a 0/1 variable.

The core scales all used a 5-point Likert scale, with 1 indicating “strongly disagree” and 5 indicating “strongly agree.” The score for each construct is the mean of its constituent items, and a higher score indicates a higher level of that variable. For established foreign scales, this study strictly followed the translation and back-translation procedure, which was conducted independently by bilingual researchers in relevant fields to ensure cross-cultural context and semantic equivalence of localized expressions. Regarding specific scales, social media use integration was contextually adapted with reference to the Facebook Intensity Scale and the Social Media Use Integration Scale, comprising five items (Ellison et al., 2007; Jenkins-Guarnieri et al., 2013). The perceived urban memory narratives scale was developed by integrating theories of urban memory, new media narratives, and Dongguan's local cultural context. The items were designed to capture migrants' perception of story-based digital content related to Dongguan's history, cultural memory, labor experience, neighborhood life, and spatial change, rather than general city news or promotional city-image information. After the initial draft was formed, the study invited 3 experts in communication and psychology to evaluate content validity, and conducted a pilot study on 50 qualified migrants in Dongguan before formal large-scale application. Based on expert opinions and participant feedback, the wording of some items was finely tuned to ensure readability for the target group, resulting in a final scale of six items. Narrative transportation was contextually adapted with reference to the Narrative Transportation Scale and its short form, comprising 8 items (Appel et al., 2015; Green and Brock, 2000). Place attachment referred to Williams and Vaske's place attachment scale, containing two dimensions: place identity and place dependence, comprising 12 items (Williams and Vaske, 2003). The urban identity scale was contextually adapted by combining classic item expressions from social identity and organizational identity research, comprising 7 items (Cameron, 2004; Mael and Ashforth, 1992). The life satisfaction scale was primarily adapted from the Satisfaction With Life Scale, contextually adapted to the specific living context in Dongguan, comprising 5 items, focusing on the life satisfaction dimension (Diener et al., 1985). Social support was contextually adapted with reference to the Multidimensional Scale of Perceived Social Support, comprising 6 items (Zimet et al., 1988). The full questionnaire items are shown in Appendix A; the variable coding names, item correspondences, scoring methods, and construct synthesis rules are shown in Appendix B.

### 3.3 Data processing and analysis methods

Before data analysis, samples not meeting the research target conditions were excluded according to questionnaire screening rules, and low-quality questionnaires were identified based on attention check items and obvious patterned responses. Multiple-choice questions were coded using the multiple-dichotomy method, splitting each option into an independent 0/1 variable. Core scales formed variable scores via item means.

The data analysis proceeded in the following steps. First, descriptive statistics were conducted to present the sample's demographic characteristics, social media use, and urban memory content access preferences. Second, common method bias and reliability were tested, composite reliability and average variance extracted were calculated, and the fit of the measurement model was evaluated through confirmatory factor analysis. Third, basic correlation structures among core variables were depicted through Pearson correlation analysis, and discriminant validity was preliminarily evaluated using the Fornell-Larcker criterion (Fornell and Larcker, 1981). Fourth, hierarchical regression was used to test the relationships among perceived urban memory narratives, narrative transportation, and place attachment, as well as the relationships among place attachment, urban identity, and life satisfaction. Fifth, the Bootstrap method was used to test the mediating association of place attachment between perceived urban memory narratives and life satisfaction. Sixth, group comparison analysis was used to test the moderating association of length of residence between perceived urban memory narratives and place attachment.

The reported model fit indices include χ^2^, *df* , χ^2^/*df* , CFI, TLI, RMSEA, and SRMR. Continuous variables in regression analyses were *Z*-score standardized before entering the models to improve the comparability of regression coefficients. Length of residence was treated as an ordinal control variable in main effect and mediation models; in the moderating association test, considering its ordinal categorical attribute, this study did not simply treat it as continuous to construct a product interaction term, but adopted a group comparison analysis strategy, dividing the sample into “3 years and below” and “more than 3 years” groups. The three-year threshold was chosen because it represents a balanced cut-off point near the sample's median residence duration, ensuring sufficient statistical power and balanced subgroup sizes (*n* = 348 vs. *n* = 542) for multi-group comparison. Additionally, this threshold aligns with local municipal integration policies in the Pearl River Delta, where a cumulative three-year residence and social security contribution history constitutes the standard eligibility baseline for migrants to access permanent public services. An inter-group slope difference test was then conducted to determine whether the association strength of perceived urban memory narratives on place attachment differed significantly between the two groups. Mediation analysis was conducted using the Bootstrap method with 5,000 resamples to test the significance of indirect associations (Hayes, 2017); if the 95% confidence interval did not contain 0, the mediating association was considered statistically significant. Because the research design is a cross-sectional survey, all statistical results are expressed in terms such as “correlation,” “association,” “mediating association,” and “moderating association,” without strict causal inferences.

## 4 Research results

### 4.1 Basic characteristics and background profile of the sample

As shown in Table 1, among the 890 valid samples recovered, male respondents accounted for a somewhat larger proportion (56.5% vs. 43.5%). In terms of age distribution, respondents aged 26–30 years (39.0%) and 31–40 years (28.0%) constituted the majority of the sample. Education levels were relatively well distributed, with associate degrees (27.1%) and bachelor's degrees or above (32.4%) accounting for nearly 60%.

**Table 1:** Basic demographic characteristics of the sample (*N* = 890).

Variable	Category	Frequency	Percentage
Gender	Male	503	56.5
	Female	387	43.5
Age	18–25 years old	174	19.6
	26–30 years old	347	39.0
	31–40 years old	249	28.0
	41 years old and above	120	13.5
Education	Junior high or below	130	14.6
	High school/Vocational	231	26.0
	Associate degree	241	27.1
	Bachelor's degree or above	288	32.4
Length of residence	6 months−1 year	71	8.0
	1–3 years	277	31.1
	3–5 years	276	31.0
	5–10 years	185	20.8
	10 years and above	81	9.1

Regarding household registration and length of residence, which measure the migrant background of respondents, 48.7% of respondents had household registration outside Guangdong Province, showing Dongguan's sample characteristics as a population-inflow city. In terms of residence length, there were 31.1% “1–3 years” residents, 31.0% “3–5 years” residents, and 20.8% “5–10 years” residents. The varied residence durations provide a sample basis for observing the relationship between the time dimension and local emotions later.

**Figure 3:**
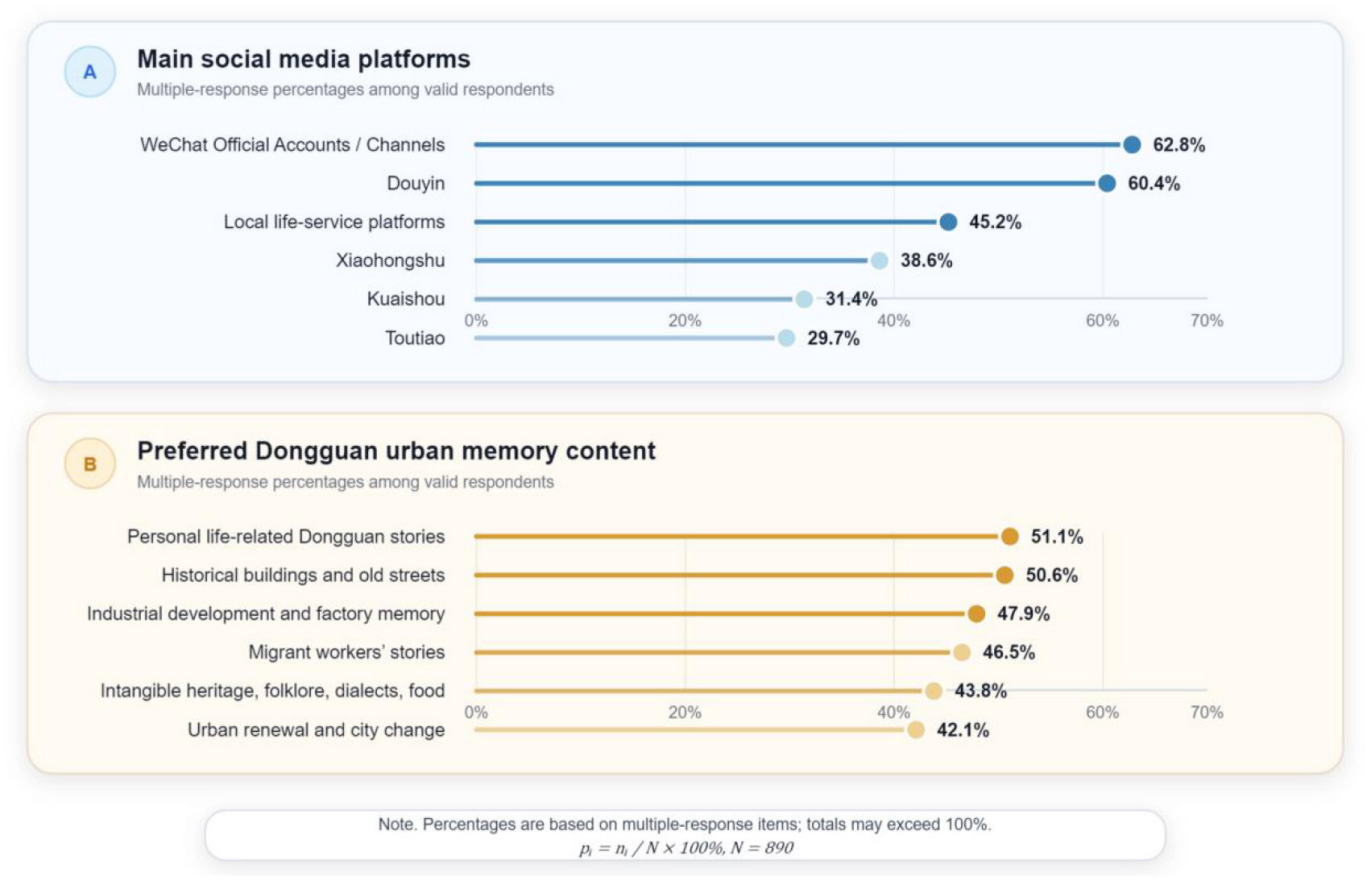
Social media platform use and preferences for Dongguan urban memory content. **(A)** Main social media platforms. Multiple-response percentages among valid respondents. **(B)** Preferred Dongguan urban memory content. Multiple-response percentages among valid respondents. Percentages in the figure are based on multiple-choice question statistics. Multiple-choice questions are coded using the multiple-dichotomy method, meaning each option is split into a 0/1 variable; therefore, the sum of percentages for each option may exceed 100%.

In terms of digital platform use, WeChat video accounts/official accounts (62.8%) and Douyin (60.4%) were important channels for respondents to access Dongguan stories. To further present respondents' digital media exposure background and preferences for Dongguan urban memory content, this study conducted multiple-dichotomy statistics on relevant multiple-choice questions, with results shown in Figure 3.

Overall, respondents' more preferred content included “Dongguan stories related to personal life” and “historical buildings and old neighborhoods.” This indicates that compared to purely macro-level promotion, life-oriented, story-based urban memory content is more likely to enter migrants' daily media exposure.

### 4.2 Reliability and validity testing of measurement instruments

Before examining relationships between variables, this study first evaluated the risk of common method bias and the reliability and validity of the measurement tools.

Given that all variables were measured using self-report questionnaires, a risk of common method bias (CMB) may exist. The study adopted Harman's single-factor test for preliminary evaluation (Podsakoff et al., 2003). After conducting an unrotated exploratory factor analysis on the 49 measurement items, the variance explained by the first factor was 25.66%, significantly lower than the 50% threshold. This suggests that common method bias is unlikely to significantly distort the relationships between variables in this study. However, Harman's single-factor test is only a preliminary diagnostic method, and the possibility of common method bias cannot be completely ruled out.

Reliability analysis showed that Cronbach's α values for all scales ranged from 0.813 to 0.906, and composite reliability (CR) values ranged from 0.870 to 0.920, indicating excellent internal consistency. Regarding convergent validity, Table 2 shows that the Average Variance Extracted (AVE) for most constructs exceeded the 0.50 threshold. For Narrative Transportation (NT) and Place Attachment (PA), the AVE scores were 0.476 and 0.491, respectively. Although these are slightly below the common 0.50 cutoff, according to Fornell and Larcker (1981), if the CR is higher than 0.60, the convergent validity of a construct may still be considered acceptable even if the AVE is lower than 0.50. Given the high CR values and the inherent complexity of measuring psychological attachment in a diverse migrant sample, these constructs were retained for further analysis.

**Table 2:** Reliability and convergent validity tests of core measurement scales.

Variable	No. of items	Cronbach's α	Composite reliability (CR)	Average variance extracted (AVE)
Social media use integration (SMU)	5	0.813	0.870	0.572
Perceived urban memory narratives (UMN)	6	0.829	0.875	0.539
Narrative transportation (NT)	8	0.842	0.879	0.476
Place attachment (PA)	12	0.906	0.920	0.491
Urban identity (UI)	7	0.839	0.879	0.509
Life satisfaction (LS)	5	0.837	0.885	0.606
Social support (SS)	6	0.853	0.891	0.576

To ascertain construct distinctiveness, this study conducted a Confirmatory Factor Analysis (CFA) based on the 49 original items. The model was specified such that each item loaded only on its theoretically assigned latent variable, and no item residuals were allowed to correlate. As shown in Table 3, the seven-factor baseline model demonstrated a better fit than the alternative models. Considering the large number of indicators (49 items) and the non-artificial model specification (no error correlations), the fit indices (χ^2^/*df* = 2.85, CFI = 0.892, TLI = 0.885, RMSEA = 0.068, SRMR = 0.058) suggest that the measurement model provides an acceptable representation of the data structure. Although CFI and TLI were slightly below the conventional 0.90 threshold, the seven-factor model showed clearly better fit than the five-factor, three-factor, and single-factor alternatives, supporting the empirical distinctiveness of the constructs.

**Table 3:** Confirmatory factor analysis (CFA) fit indices comparison.

Model specification	χ^2^	*df*	χ^2^/*df*	CFI	TLI	RMSEA	SRMR
Seven-factor baseline model	3,152.10	1,106	2.85	0.892	0.885	0.068	0.058
Five-factor model	4,977.00	1,117	4.45	0.792	0.778	0.085	0.076
Three-factor model	7,211.50	1,124	6.41	0.671	0.653	0.114	0.091
Single-factor model	11,246.30	1,127	9.98	0.455	0.421	0.162	0.125

CFA was conducted on the 49 original items without item parceling or artificial error correlation adjustments. While thresholds like CFI > 0.90 are commonly used, slightly lower values may still be considered acceptable in complex psychological models with many observed indicators (Hair et al., 2006; Marsh et al., 2004).

Finally, discriminant validity was supported, as the square root of the AVE for each construct exceeded its correlation with all other constructs. The highest inter-construct correlation was between place attachment and urban identity (*r* = 0.548), which remained comfortably below the square root of their respective AVEs, specifically, the square root of AVE for PA is 0.701 and for UI is 0.713. This result also indicates that place attachment and urban identity are related but empirically distinguishable constructs.

### 4.3 Descriptive statistics and correlation matrix of core variables

As shown in Table 4, under the five-point Likert scale, the mean scores of the core constructs were above the midpoint. Social support (*M* = 3.58), social media use integration (*M* = 3.56), and urban identity (*M* = 3.56) scored relatively high, indicating that migrant respondents have already formed a certain foundation of interpersonal support, media embeddedness, and identity acceptance in Dongguan.

**Table 4:** Means, standard deviations, and correlation matrix of core variables (*N* = 890).

Variable	*M*	*SD*	1	2	3	4	5	6	7	8
1. Social media use integration (SMU)	3.56	0.83	1							
2. Perceived urban memory narratives (UMN)	3.53	0.81	0.404^***^	1						
3. Narrative transportation (NT)	3.55	0.75	0.313^***^	0.457^***^	1					
4. Place attachment (PA)	3.55	0.78	0.313^***^	0.336^***^	0.456^***^	1				
5. Urban identity (UI)	3.56	0.78	0.329^***^	0.386^***^	0.415^***^	0.548^***^	1			
6. Life satisfaction (LS)	3.50	0.87	0.214^***^	0.310^***^	0.315^***^	0.473^***^	0.439^***^	1		
7. Social support (SS)	3.58	0.84	0.213^***^	0.263^***^	0.313^***^	0.426^***^	0.365^***^	0.400^***^	1	
8. Length of residence (Q4, ordinal coding)	2.92	1.09	0.023	0.032	−0.014	0.006	0.035	−0.004	0.034	1

Values below the diagonal are Pearson correlation coefficients; ^***^p < 0.001. Length of residence is an ordinal categorical variable; here, its descriptive statistics and correlation results after coding are reported. Length of residence is coded as 1 = 6 months−1 year, 2 = 1–3 years, 3 = 3–5 years, 4 = 5–10 years, 5 = 10 years and above.

Pearson correlation analysis preliminarily depicted the relationship structure among variables. Results showed a significant positive correlation between social media use integration and perceived urban memory narratives (*r* = 0.404, *p* < 0.001), indicating that respondents whose daily lives were more embedded in social media were more likely to access and perceive Dongguan urban memory-related content. Perceived urban memory narratives, narrative transportation, and place attachment were all significantly positively correlated with each other. At the same time, a strong positive correlation was also observed between place attachment and life satisfaction (*r* = 0.473, *p* < 0.001). Urban identity was significantly positively correlated with both place attachment (*r* = 0.548, *p* < 0.001) and life satisfaction (*r* = 0.439, *p* < 0.001), providing preliminary support for its explanatory value as a psychological integration variable.

### 4.4 Hypothesis testing: regression, mediation, and moderation model analysis

To test the six hypotheses proposed in this paper, this study conducted model testing while controlling for demographic variables and relevant background variables. Except for the length of residence grouping analysis model, the core models mainly controlled for gender, age, education level, monthly income, length of residence, social media use integration, and social support; in the length of residence grouping model, length of residence was used as the grouping basis and no longer included as a control variable in the regression equation. Continuous variables or ordinal control variables entering the regression models were *Z*-score standardized to ensure comparability across coefficients. Gender was dummy-coded, with male = 0 and female = 1.

#### 4.4.1 Urban memory narratives, narrative transportation, and place attachment: testing H1 and H2

As shown in Table 5, to test H1 and H2, this study established a hierarchical regression model with place attachment (PA) as the dependent variable. Model 1 included control variables such as gender, age, education level, monthly income, length of residence, social media use integration, and social support; the model explained 23.7% of the variance in place attachment. Model 2 added perceived urban memory narratives (UMN) based on the control variables. Results showed that UMN was significantly and positively associated with place attachment (β = 0.177, *p* < 0.001), and the model's explanatory power increased by 2.5%. Thus, H1 is supported.

**Table 5:** Hierarchical regression models with place attachment (PA) as dependent variable.

Predictor variables	Model 1 β	Model 2 β	Model 3 β
Control variables			
Gender (Q6)	−0.017	−0.015	−0.005
Age (Q2)	0.043	0.035	0.028
Education level (Q7)	0.035	0.035	0.046
Monthly income (Q12)	−0.026	−0.034	−0.025
Length of residence (Q4)	−0.017	−0.019	−0.009
Social media use integration (SMU)	0.235^***^	0.170^***^	0.131^***^
Social support (SS)	0.375^***^	0.343^***^	0.286^***^
Explanatory variables			
Perceived urban memory narratives (UMN)	—	0.177^***^	0.074^*^
Narrative transportation (NT)	—	—	0.291^***^
Model fit indices			
*R* ^2^	0.237	0.262	0.325
Adjusted *R*^2^	0.231	0.256	0.318
Δ*R*^2^	—	0.025^***^	0.063^***^
*F*-value	39.23^***^	39.20^***^	46.98^***^

Regression coefficients are standardized coefficients β; “—” indicates the variable was not included in the corresponding model.

^*^p < 0.05, ^***^p < 0.001.

Model 3 further added narrative transportation (NT). Results showed that narrative transportation was significantly and positively associated with place attachment (β = 0.291, *p* < 0.001), and the model's explanatory power further increased by 6.3%. Thus, H2 is supported. At this point, the coefficient for UMN was still significant (β = 0.074, *p* < 0.05), but lower than in Model 2, indicating that the relationship between perceived urban memory narratives and place attachment is partially intertwined with narrative transportation experience. Although the reduction in the UMN coefficient (from β = 0.177 to β = 0.074) hints at a potential mediating or channeling role of narrative transportation, we chose a parallel hierarchical regression approach rather than a multi-stage serial mediation model. This choice was made to avoid the risk of statistical overfitting and to maintain a conservative modeling strategy appropriate for cross-sectional data.

#### 4.4.2 Place attachment, urban identity, and life satisfaction: testing H3, H5a, and H5b

As shown in Table 6, to test the relationships among place attachment, urban identity, and life satisfaction, this study constructed a hierarchical regression model with life satisfaction (LS) as the dependent variable. Model 1 included control variables such as gender, age, education level, monthly income, length of residence, social media use integration, and social support. Results showed that social support was significantly and positively associated with life satisfaction (β = 0.374, *p* < 0.001), and social media use integration was also significantly and positively associated with life satisfaction (β = 0.136, *p* < 0.001). The model explained a total of 18.4% of the variance in life satisfaction.

**Table 6:** Hierarchical regression models with life satisfaction (LS) as dependent variable.

Predictor variables	Model 1 β	Model 2 β
Control variables		
Gender (Q6)	−0.082^**^	−0.078^**^
Age (Q2)	0.000	−0.010
Education level (Q7)	−0.013	−0.022
Monthly income (Q12)	0.013	0.035
Length of residence (Q4)	−0.020	−0.021
Social media use integration (SMU)	0.136^***^	0.018
Social support (SS)	0.374^***^	0.209^***^
Explanatory variables		
Place attachment (PA)	—	0.261^***^
Urban identity (UI)	—	0.218^***^
Model fit indices		
*R* ^2^	0.184	0.311
Adjusted *R*^2^	0.178	0.305
Δ*R*^2^	—	0.127^***^
*F*-value	28.50^***^	44.29^***^

Regression coefficients are standardized coefficients β; “—” indicates the variable was not included in the corresponding model.

^**^p < 0.01, ^***^p < 0.001.

Subsequently, place attachment (PA) and urban identity (UI) were simultaneously introduced in Model 2. Results showed that after controlling for demographic variables, length of residence, social media use integration, and social support, place attachment maintained a significant positive association with life satisfaction (β = 0.261, *p* < 0.001), indicating that the higher the level of place attachment, the more positive respondents' overall evaluation of their current living status in Dongguan. Thus, H3 is supported.

At the same time, urban identity was also significantly and positively associated with life satisfaction (β = 0.218, *p* < 0.001). This indicates that urban identity has an independent positive statistical association with life satisfaction. With the addition of place attachment and urban identity, the overall explanatory power of Model 2 increased to 31.2%, an improvement of 12.7 percentage points over the baseline model (Δ*R*^2^ = 0.127, *p* < 0.001).

Combining the previous correlation analysis, it can be seen that there is a significant positive correlation between urban identity and place attachment (*r* = 0.548, *p* < 0.001). This confirms the expected positive correlation between the two integration dimensions while supporting their empirical distinctiveness. Meanwhile, regression results showed that after controlling for demographic variables, length of residence, social media use integration, social support, and place attachment, urban identity was still significantly and positively associated with life satisfaction (β = 0.218, *p* < 0.001); therefore, H5 is supported.

#### 4.4.3 Mediating association of place attachment: testing H4

To further examine the indirect association of place attachment between perceived urban memory narratives and life satisfaction, this study employed the Bootstrap method for mediation testing, resampling 5,000 times. The model simultaneously controlled for gender, age, education level, monthly income, length of residence, social media use integration, and social support.

As shown in Table 7, results showed that perceived urban memory narratives were significantly and positively associated with place attachment [*a* = 0.177, 95% CI (0.113, 0.239)], and place attachment was significantly and positively associated with life satisfaction [*b* = 0.330, 95% CI (0.269, 0.391)]. The total association of perceived urban memory narratives on life satisfaction was 0.196, the direct association was 0.138, and the indirect effect was 0.058, with a 95% Bootstrap confidence interval of [0.036, 0.083], which did not contain 0. This result indicates that after controlling for relevant background variables, place attachment presented a partial mediating relationship between perceived urban memory narratives and life satisfaction, with the indirect association accounting for approximately 29.6% of the total association. It is worth pointing out that since this study is a cross-sectional design, this mediation result only indicates an indirect statistical association consistent with theoretical expectations between the variables, and strict causal processes cannot be inferred from this. H4 is supported.

**Table 7:** Bootstrap mediation model after controlling for relevant background variables.

Path/Effect	Effect estimate	Bootstrap *SE*	95% Bootstrap CI	Proportion
Path *a*: UMN → PA	0.177^***^	0.032	[0.113, 0.239]	—
Path *b*: PA → LS	0.330^***^	0.031	[0.269, 0.391]	—
Total effect *c*	0.196^***^	0.031	[0.135, 0.259]	100%
Direct effect *c*'	0.138^***^	0.032	[0.076, 0.201]	70.4%
Indirect effect *a × b*	0.058	0.012	[0.036, 0.083]	29.6%

The model controlled for gender, age, education level, monthly income, length of residence, social media use integration, and social support. Bootstrap resampling was set to 5,000 times. The confidence interval of the indirect effect did not contain 0, indicating that the mediating association reached statistical significance.

^***^p < 0.001.

**Figure 4:**
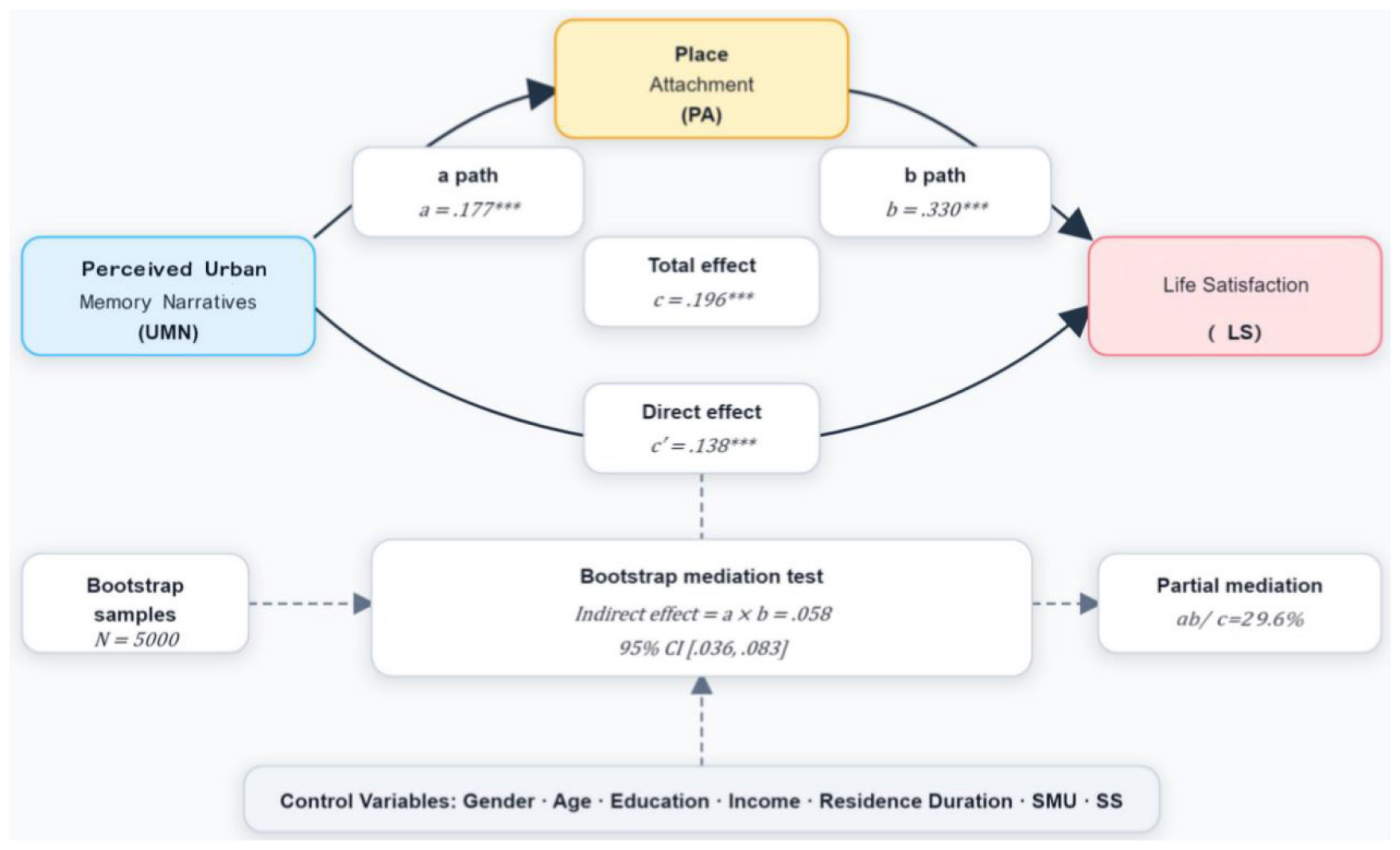
Path diagram of the mediating association of place attachment. UMN, Perceived social media urban memory narratives; PA, Place attachment; LS, life satisfaction; SMU, Social media use integration; SS, Social support. Path coefficients in the figure are from the Bootstrap mediation model after controlling for gender, age, education level, monthly income, length of residence, SMU, and SS. Bootstrap resampling was 5,000 times. ***p < 0.001.

To intuitively present the mediating association of place attachment between perceived urban memory narratives and life satisfaction, this paper further provides a path diagram of the Bootstrap mediation test results, specifically as shown in Figure 4.

#### 4.4.4 Moderating association of length of residence: testing H6

Considering that length of residence is essentially an ordinal categorical variable, using a traditional continuous variable product term for moderation testing might carry the statistical risk of invalid strict linear assumptions. To more accurately explore the boundary condition of length of residence, this study adopted a multi-group analysis approach, recoding and dividing length of residence into two major subgroups: combining “6 months−1 year” and “1–3 years” into the “3 years and below” group (*n* = 348, accounting for 39.1%), representing newly arrived migrants in the initial integration period; and combining “3–5 years,” “5–10 years,” and “10 years and above” into the “more than 3 years” group (*n* = 542, accounting for 60.9%), representing the longer-residing group in a stable residence period.

After controlling for gender, age, education level, monthly income, social media use integration, and social support, regression analysis with place attachment (PA) as the dependent variable was conducted separately on the two groups using standardized data. As shown in Table 8, perceived urban memory narratives (UMN) had a significant positive association with place attachment in the newly arrived group of “3 years and below” (β = 0.194, *p* < 0.001); it also showed a significant positive association in the longer-residing group of “more than 3 years” (β = 0.162, *p* < 0.001). Further inter-group slope difference testing showed that the difference between the regression coefficients of the two groups did not reach statistical significance (*Z* = 0.484, *p* = 0.629). Therefore, the directional pattern was consistent with H6, but the difference was not statistically significant; H6 is not supported.

**Table 8:** Regression models of perceived urban memory narratives on place attachment by length of residence groups.

Predictor variables	“3 years and below” Group (*n* = 348) β	“More than 3 years” Group (*n* = 542) β
Control variables		
Gender (Q6)	0.018	−0.034
Age (Q2)	20.020	0.067
Education level (Q7)	0.048	0.026
Monthly income (Q12)	0.003	−0.061
Social media use integration (SMU)	0.182^***^	0.160^***^
Social support (SS)	0.342^***^	0.346^***^
Core predictor variable		
Perceived urban memory narratives (UMN)	0.194^***^	0.162^***^
Model fit indices		
*R* ^2^	0.294	0.246
Adjusted *R*^2^	0.280	0.236
*F-*value	20.24^***^	24.86^***^

The dependent variable is place attachment (PA). The table presents standardized regression coefficients β after controlling for background variables. The inter-group coefficient difference test did not reach statistical significance (Z = 0.484, p > 0.05).

^***^p < 0.001.

**Figure 5:**
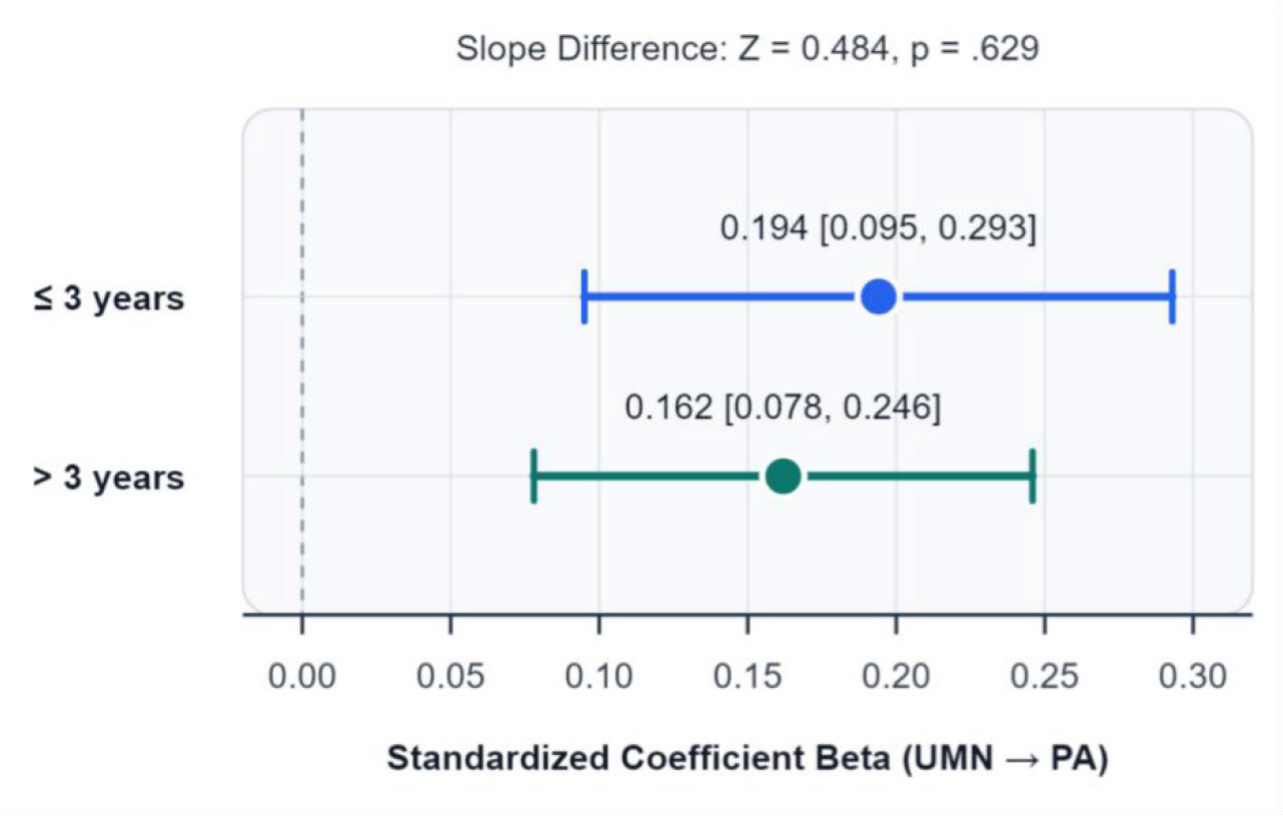
Comparison of regression coefficients for the association between perceived urban memory narratives and place attachment by length of residence groups. Points in the figure represent the standardized regression coefficients of perceived urban memory narratives (UMN) on place attachment (PA), and error bars indicate 95% confidence intervals. The models controlled for gender, age, education level, monthly income, social media use integration, and social support. Inter-group slope difference test did not reach statistical significance (*Z* = 0.484, *p* = 0.629).

To more intuitively display the closeness of core path coefficients in different length of residence groups, this paper further plotted the standardized regression coefficients of perceived urban memory narratives (UMN) on place attachment (PA) and their 95% confidence intervals as Figure 5. This figure only shows the core predictor variable results from Table 8 and does not redundantly present control variables and model fit indices.

## 5 Discussion and practical implications

Because this study relies on cross-sectional data, all discussed links represent statistical associations rather than causal effects, and the possibility of reverse causality—where individuals with high baseline place attachment or satisfaction are more receptive to local media narratives—cannot be ruled out. Furthermore, the outcome construct is operationalized specifically through life satisfaction.

### 5.1 The relationship between social media urban memory narratives and place attachment

The results of this study show that there is a significant positive statistical association between perceived social media urban memory narratives and migrants' place attachment. After controlling for demographic variables, social media use integration, and social support, perceived urban memory narratives still maintained a significant relationship with place attachment. This result suggests that migrants' emotional connection to the city is not only related to years of residence, social support, or economic conditions, but also linked to the urban narratives they are exposed to in the digital media environment, although individuals with higher pre-existing place attachment might also actively select and notice such narratives. Indeed, a self-reinforcing loop may exist where initial local attachment drives active engagement with Dongguan-related online content.

This finding is of great significance for understanding the urban experience of migrants in Dongguan. For many migrants, the initial motivation for coming to Dongguan is quite realistic; job opportunities, income expectations, living costs, and social networks are often their primary considerations for entering the city. Urban cultural cognition and local emotion are not necessarily the starting point of moving in, but gradually form during work, residence, commuting, consumption, and media exposure. Urban memory narratives on social media may provide a low-threshold, everyday path for this emotional formation, which also echoes views on digital placemaking and short video local narratives regarding digital media's participation in constructing place meaning (Li and Alencar, 2023; Liu and Zhang, 2024). This theoretical extension is particularly crucial because traditional place attachment research has predominantly focused on physical presence and offline social capital. Our findings suggest that in high-mobility digital societies, symbolic mediated interactions on platforms can serve as a vital virtual extension of the physical environment, thus expanding the boundaries of place-attachment theory to include digital placemaking practices.

Compared with traditional urban propaganda, social media urban memory narratives are more likely to present the city in a life-oriented and story-based way. Changes in old neighborhoods, factory memories, urban village life, intangible cultural food, stories of ordinary workers, and community renewal experiences may all enter the information flow of migrants through short videos, graphic notes, or local official accounts. For migrants, such content may not directly change their living conditions, but it may help them re-understand the relationship between themselves and the city. Therefore, the communication of Dongguan's urban memory should not stop at the level of macro-achievements, city image, and industrial labels, but should present more micro-narratives that can connect with personal life, turning the city image from a “displayed Dongguan” to an “experienced Dongguan.”

### 5.2 The psychological significance of narrative transportation

The research results also show that narrative transportation and place attachment have a significant positive association. In the hierarchical regression model, after adding narrative transportation, the model's explanatory power further improved, and narrative transportation was significantly and positively correlated with place attachment. This suggests that respondents do not just passively receive urban memory information; the attentional focus, scene imagination, emotional involvement, and continuous reflection they form while watching or reading related content may be an important psychological correlate—rather than a confirmed causal driver—for understanding place attachment (Appel et al., 2015; Green and Brock, 2000).

Methodologically, the significant drop in the coefficient of urban memory narratives when narrative transportation was entered into the model suggests that the cognitive-emotional immersion of media reception serves as a critical mechanism linking message exposure to spatial emotions. From a digital placemaking perspective, this implies that merely encountering urban content online is insufficient; it is the subjective depth of psychological transport (narrative transportation) that effectively translates mediated space into a meaningful place. However, due to the inherent limits of cross-sectional surveys in verifying temporal sequences, we refrained from modeling this as a formal sequential mediation. Future longitudinal research would be highly valuable in formally testing whether narrative transportation initiates and tracks the chronological development of place attachment over time.

Compared with pure information introduction, narrative content has a stronger sense of entering a scene. If a video about the transformation of an old industrial area in Dongguan merely lists time nodes and development data, it might mostly remain at the knowledge level; if specific characters, spatial details, life changes, and emotional expressions are added, it is much easier for the audience to generate imagination and resonance. For migrants, this immersive experience is particularly noteworthy. An unfamiliar city is not just strange in space, but also contains cultural, identity, and emotional distance. Narrative content may relate to a lower-friction cognitive pathway for migrants adapting to the city, allowing individuals to construct mental models of the city's past and present prior to or alongside direct physical engagement.

This result has implications for urban communication practices. The content of Dongguan's urban memory should not only pursue information completeness and image unity, but also emphasize narrative structure and situational creation. For example, urban memory content can start from specific scenes such as ordinary workers, community life, industrial memories, urban renewal, and local food, making it easier for migrants to generate the psychological feeling that “this story is related to me.”

### 5.3 Place attachment, urban identity, and life satisfaction

This study further found a significant positive statistical relationship between place attachment and life satisfaction. It should be noted that the outcome variable measured in this paper is life satisfaction, so related discussions largely center around respondents' overall satisfaction with their current life in Dongguan. After controlling for variables such as demographic characteristics, social media use integration, and social support, place attachment still maintained a significant association with life satisfaction. This suggests that the sense of closeness, meaning, and functional dependence that migrants form toward Dongguan are closely related to their overall evaluation of their current living status. This result is consistent with discussions in place attachment research regarding the relationship among place identity, place dependence, and life satisfaction (Pai et al., 2024; Williams and Vaske, 2003).

For migrants, life satisfaction does not just come from income levels, housing conditions, and public services, but is also related to whether they feel they “have a place here.” Whether a person can form familiar spatial routes, dependable interpersonal relationships, a stable life rhythm, and a sense of emotional belonging in the city will all enter into their life satisfaction judgments. Place attachment is exactly the psychological expression of this “I relate to the city.” Existing research on Chinese migrants also finds that urban perception, social relationships, and place attachment are closely associated with migrants' urban integration, settlement intentions, and sense of urban belonging (Hu et al., 2024; Lu et al., 2024; Xie and Huang, 2022). When migrants no longer just see Dongguan as a temporary workspace but gradually understand it as a place with life meaning, their life satisfaction is also more likely to present a positive state.

Urban identity also plays an important role. Research results showed that urban identity had a high-level positive correlation with place attachment and a significant positive statistical relationship with life satisfaction. Place attachment emphasizes more the emotional and functional link between people and places, while urban identity highlights identity belonging and a sense of membership. Although the two constructs are closely related, discriminant validity analysis confirms that they are empirically distinguishable dimensions of integration. This empirical distinction aligns with the theoretical view that place attachment primarily reflects a spatial-functional bond, whereas urban identity represents a social-categorical group membership. By demonstrating that both variables independently relate to life satisfaction, our study highlights that migrants' integration is a multidimensional process involving both spatial adaptation and community identification, with neither being redundant.

This finding suggests that urban governance and urban communication should not only focus on service supply and image output, but also on migrants' identity recognition and emotional inclusion. For a migrant-receiving city such as Dongguan, “new citizens” should not just be management targets or service recipients, but should also be co-producers of urban memory. If urban memory narratives can better incorporate migrants' labor experiences, residential experiences, family stories, and community participation experiences, it may help translate urban identity from abstract slogans into concrete life experiences.

### 5.4 The mediating association of place attachment

The Bootstrap mediation test showed that after controlling for demographic variables, social media use integration, and social support, place attachment presented a partial mediating association between perceived urban memory narratives and life satisfaction. This result indicates that there is not only a direct statistical relationship between perceived social media urban memory narratives and life satisfaction; place attachment is one of the important psychological links bridging the two.

It must be emphasized that this result does not imply that social media content can directly increase life satisfaction. A more cautious understanding is that perceived urban memory narratives may have an indirect statistical association with life satisfaction through the sense of closeness, meaning, and functional dependence respondents form toward Dongguan. In other words, the reason social media urban stories are correlated with life satisfaction may not simply be because the content itself is pleasing, but because this content helps the audience re-understand their relationship with the city.

This also provides a new entry point for understanding migrants' psychological integration. Existing studies often link migrants' life satisfaction and broader wellbeing to income, social security, the household registration system, housing conditions, and employment stability, which are certainly very important. But this study shows that soft experiences such as local culture, urban memory, and everyday narratives also have an observable statistical relationship with place attachment and life satisfaction. If urban governance only emphasizes resource supply and ignores emotional connections in urban narratives, it may be difficult to fully understand the psychological integration process of new citizens.

At the practical level, Dongguan can combine urban memory communication with new citizen services, community building, and public cultural activities. Specifically, local organizations should move away from generic city-branding campaigns and instead co-create targeted digital storytelling initiatives with migrants. These may include localized oral history projects and worker-centered short-form videos that represent the actual labor and housing spaces (such as industrial parks and urban villages) inhabited by migrants, as identified by our survey preferences.

### 5.5 Explanation for the non-significant moderating association of length of residence

Contrary to the research hypothesis, this study did not observe a significant moderating association of length of residence between perceived urban memory narratives and place attachment. Based on multi-group analysis results, perceived urban memory narratives were significantly and positively associated with place attachment in both the “3 years and below” group and the “more than 3 years” group, but the difference in regression slopes between the two groups did not reach statistical significance. This implies that in this sample, the relationship between perceived urban memory narratives and place attachment did not show obvious changes in strength due to differences in years of residence.

This result needs to be interpreted cautiously. It does not mean that length of residence is unimportant for migrants' urban experience. In fact, residence time may still affect an individual's social network, spatial familiarity, job stability, and access to public services. Existing migrant studies also indicate that residence experience, social relationships, and urban integration status are closely associated with place attachment, settlement intentions, and sense of urban belonging (Hu et al., 2024; Lu et al., 2024; Xie and Huang, 2022). It is just that regarding the “perceived social media urban memory narratives - place attachment” relationship focused on in this study, length of residence did not show a significant boundary moderating association.

One possible explanation is that social media urban memory narratives possess a certain cross-stage penetrability. For migrants who have recently arrived in Dongguan, social media content may help them quickly understand the city; for those who have lived there longer, related content may also reactivate their existing life experiences and urban memories. That is to say, digital media does not merely serve the city initiation of “newcomers,” but may also become a trigger for long-term residents to re-understand urban changes and personal experiences.

Another possible explanation is that length of residence alone may not be sufficient to capture the heterogeneity of migrants' urban integration experiences. Two individuals with the same residence duration may differ greatly in employment stability, family migration status, neighborhood participation, housing conditions, and local social networks. Therefore, the absence of a significant moderating association may reflect the complexity of residence experience rather than the irrelevance of time.

However, this explanation still needs further verification by follow-up studies. Length of residence was treated as an ordinal categorical variable in this study, which might not fully capture the complexity of residence experience. Future research could use longitudinal designs, latent class analysis, or more granular measurements of residence experience to further examine whether there are differences between media experiences and local emotions across different residence stages.

Overall, the non-significant moderating association is still theoretically meaningful. It suggests that social media urban memory narratives may be associated with place attachment across different residence stages, rather than only among newly arrived migrants or only among long-term residents. For urban communication practice, this means that Dongguan's urban memory narratives should not be designed exclusively for one residence group. Instead, they can simultaneously provide orientation for newcomers and memory activation for longer-term migrants.

## 6 Research limitations, future directions, and conclusions

### 6.1 Research limitations and future directions

This study was conducted based on cross-sectional survey data, which can present statistical associations among perceived social media urban memory narratives, place attachment, urban identity, and life satisfaction, but cannot confirm chronological sequences between variables. Therefore, the results regarding mediating associations in this paper should be understood as indirect statistical relationships within cross-sectional data, rather than strict causal processes. Specifically, the presence of reverse causality remains a key limitation: migrants who are already highly attached to Dongguan or generally satisfied with their lives may be more predisposed to actively seek out, perceive, and immerse themselves in local urban narratives on social media. Migrants' local emotions are often interwoven with work, residence, socializing, media exposure, and everyday life experiences; future research could use tracking surveys, longitudinal designs, or phased interviews to more meticulously observe the changing process of their urban experiences and local emotions. Furthermore, while our results hint at a potential channeling role of narrative transportation, the cross-sectional design prevented us from formally modeling a sequential mediation chain (narrative perception → narrative transportation → place attachment → life satisfaction). Future studies should employ longitudinal or experimental designs to formally verify whether narrative transportation serves as the chronological psychological agent that translates media narratives into spatial attachment over time.

The data in this study mainly come from self-reported questionnaires and may therefore be affected by recall bias, social desirability bias, or respondents' current emotional states. Additionally, the survey relied on online anonymous convenience sampling, resulting in a non-probability sample that may not fully represent all internal migrants in Dongguan, particularly those with limited digital literacy. Furthermore, the broad categorization of “internal migrants” obscures the high socioeconomic and occupational heterogeneity within this population, such as the distinct lifestyles and adaptation barriers of blue-collar factory workers compared to high-tech white-collar professionals. Future studies must incorporate representative sampling designs and subgroup analyses to examine how varying socio-demographic backgrounds affect these media-psychology dynamics. Although the study controlled and evaluated data quality and common method bias risk through screening questions, attention checks, exclusion of low-quality samples, Harman's single-factor test, and CFA model comparisons, the limitations of homologous measurements are still hard to entirely eliminate. Social media use itself has strong contextual attributes, and different platforms, content forms, and usage habits might correspond to different psychological experiences. Subsequent research could combine in-depth interviews, platform texts, comment data, or digital ethnography methods to further understand how migrants access, interpret, and respond to urban memory content.

The research field is focused on Dongguan, which allows this paper to conduct a relatively specific discussion around a typical population-inflow manufacturing city, but also limits the extrapolation scope of the conclusions. Dongguan's industrial structure, population composition, urban memory resources, and media ecology have their own characteristics and should not directly represent all migrant cities. Future studies could include population-inflow cities such as Guangzhou, Shenzhen, Foshan, Suzhou, and Ningbo in comparative research to observe whether the association between social media urban memory narratives and place attachment presents a similar structure under different urban contexts.

In addition, this paper treated social media urban memory narratives as an aggregate variable, without yet subdividing types such as official narratives, self-media narratives, industrial memories, intangible cultural heritage content, urban renewal narratives, and ordinary workers' stories. Different narrative subjects and content styles might present different associations with audiences' local emotions. Life satisfaction in this paper was also primarily measured as the cognitive dimension of subjective wellbeing, without fully covering broader dimensions such as positive emotions, psychological security, or a sense of life meaning. Future research could further refine content types and psychological wellbeing measurements while keeping the questionnaire length manageable.

Length of residence was treated as an ordinal categorical variable and grouping variable in this study. This approach helps avoid the linear assumption problems brought by simple continuous scaling, but still cannot fully present the complexity of residence experience. Migrants with the same years of residence might have distinct differences in job stability, family relocation, community participation, and social relationships, which may introduce unobserved heterogeneity into the moderating analysis. Subsequent research could incorporate more detailed and multidimensional residence experience indicators to better understand the relationship between media experience and local emotions under different living contexts.

### 6.2 Conclusion

Taking migrants in Dongguan as subjects and based on 890 valid cross-sectional questionnaires, this study examined the statistical relationships among perceived social media urban memory narratives, narrative transportation, place attachment, urban identity, and life satisfaction. Results showed that perceived social media urban memory narratives were significantly and positively associated with place attachment, and narrative transportation was also significantly and positively associated with place attachment; both place attachment and urban identity were significantly and positively associated with life satisfaction; place attachment presented a partial mediating association between perceived urban memory narratives and life satisfaction; the moderating association of length of residence did not reach a significant level, but the positive association between perceived urban memory narratives and place attachment remained consistent across different length of residence groups.

These results suggest that, as digital media becomes increasingly embedded in everyday life, urban memory is not merely cultural material for city image communication but also an empirical clue for understanding migrants' urban experiences. For a migrant-receiving city such as Dongguan, the way many migrants initially encounter the city is often related to work, residence, and living costs, and local emotion is not necessarily formed at the beginning of moving in. Urban memory narratives on social media, especially content related to ordinary people's lives, urban changes, labor experiences, and neighborhood spaces, may present a certain association with their understanding of and emotional connection to Dongguan. The city is not an object to be known all at once, but a living space gradually understood through residing, watching, narrating, interacting, and participating. Urban memory narratives on social media are an important entry point for observing this process.

## Data Availability

The original contributions presented in the study are included in the article/Supplementary material, further inquiries can be directed to the corresponding author.
