# Supplementary material for: Urban memory narratives on social media, place attachment among internal migrants, and life satisfaction: a case study of Dongguan
Source: Front Psychol. 2026 Jul 21;17:1887411. doi: 10.3389/fpsyg.2026.1887411 (PMC13433500; doi:10.3389/fpsyg.2026.1887411)
Supplement: Supplementary file 1 [file Data_Sheet_1.zip › Appendix_B_Clean_Data.docx]

Appendix B: Variable Codebook

**I. Core Scale Variables**

The following matrix scale questions all use a 5-point Likert scoring: 1=Strongly Disagree, 2=Disagree, 3=Neutral, 4=Agree, 5=Strongly Agree.

| Variable Prefix and Range | Representative Construct (Indicator) | No. of Items | Corresponding Questionnaire Module |
| --- | --- | --- | --- |
| SMU (SMU1 - SMU5) | Social Media Use Integration | 5 | Q20 |
| UMN (UMN1 - UMN6) | Perceived Social Media Urban Memory Narratives | 6 | Q21 |
| NT (NT1 - NT8) | Narrative Transportation | 8 | Q22 |
| PA (PA1 - PA12) | Place Attachment (PA1-PA6 are Place Identity, PA7-PA12 are Place Dependence) | 12 | Q23 |
| UI (UI1 - UI7) | Urban Identity | 7 | Q24 |
| SWB (SWB1 - SWB5) | Subjective Well-being (Life Satisfaction Dimension) | 5 | Q25 |
| SS (SS1 - SS6) | Social Support | 6 | Q26 |

Note: When conducting tests such as correlation and regression, the score for each construct is generated by taking the mean of its corresponding items.

**II. Single-choice Categorical/Ordinal Variables**

Includes screening questions, demographic characteristics, and basic usage conditions.

| Variable Name | Meaning | Value and Coding |
| --- | --- | --- |
| Q1 | Informed Consent | 1=Agree; 2=Disagree (All 1 in the data) |
| Q2 | Age | 1=Under 18; 2=18-25; 3=26-30; 4=31-40; 5=41-50; 6=Over 51 |
| Q3 | Whether in Dongguan | 1=Yes; 2=No (All 1 in the data) |
| Q4 | Time in Dongguan | 1=Less than 6 months; 2=6 months-1 year; 3=1-3 years; 4=3-5 years; 5=5-10 years; 6=Over 10 years |
| Q5 | Hukou Distinction | 1=Dongguan local long-term resident; 2=Migrant to Dongguan (All 2 in the data) |
| Q6 | Gender | 1=Male; 2=Female; 3=Other |
| Q7 | Education | 1=Junior high and below; 2=High school/Vocational; 3=Associate degree; 4=Bachelor’s; 5=Master’s and above |
| Q8 | Marital Status | 1=Unmarried; 2=Married; 3=Divorced/Separated; 4=Widowed; 5=Other |
| Q9 | Occupational Identity | 1=Enterprise/Factory worker; 2=Service industry; 3=Self-employed/Freelancer; 4=Company staff; 5=Professional and technical; 6=Student; 7=No fixed employment; 8=Other |
| Q10 | Hukou Location | 1=Dongguan Hukou; 2=Non-Dongguan Hukou within province; 3=Out-of-province Hukou; 4=Other |
| Q11 | Living Arrangement | 1=Owned housing; 2=Rented housing; 3=Company/Factory dorm; 4=Living with family/friends; 5=Other |
| Q12 | Monthly Income | 1=Below 3000 RMB; 2=3000-4999 RMB; 3=5000-7999 RMB; 4=8000-11999 RMB; 5=12000 RMB and above; 6=Prefer not to say |
| Q13 | Intention to Stay | 1=None; 2=Unsure; 3=Some intention; 4=Relatively clear; 5=Very clear |
| Q14 | Usage Frequency | 1=Almost never; 2=1-2 times/week; 3=3-5 times/week; 4=Almost every day; 5=Multiple times a day |
| Q15 | Daily Duration | 1=Less than 30 mins; 2=30 mins-1 hour; 3=1-2 hours; 4=2-4 hours; 5=Over 4 hours |
| Q17 | Exposure Frequency | 1=Often see; 2=Occasionally see; 3=Rarely see; 4=Almost never see; 5=Not sure |
| ATT1 | Quality Control Check | Q27 Attention check question (Value 4 is pass, all 4 in the final cleaned data) |

**III. Multiple-choice Variables (Binary Split)**

The data file splits the multiple-choice questions in the questionnaire into independent binary variables. Uniform coding rule: 0=Not selected, 1=Selected.

| Split Variable Group | Corresponding Q No. | Measurement Content | Brief Description of Variables |
| --- | --- | --- | --- |
| Q16_A ~ Q16_K | Q16 | Frequently used platforms | A=Douyin, B=Kuaishou, C=WeChat, D=Xiaohongshu … K=Almost do not use |
| Q18_A ~ Q18_K | Q18 | Form of exposure | A=Short video, B=Graphic/text, C=Live stream, D=Comments ... J=Almost no contact |
| Q19_A ~ Q19_J | Q19 | Content followed | A=Historical buildings, B=Industrial memory, C=Struggle stories … I=Do not pay attention |
