# Supplementary material for: Urban memory narratives on social media, place attachment among internal migrants, and life satisfaction: a case study of Dongguan
Source: Front Psychol. 2026 Jul 21;17:1887411. doi: 10.3389/fpsyg.2026.1887411 (PMC13433500; doi:10.3389/fpsyg.2026.1887411)
Supplement: Supplementary file 1 [file Data_Sheet_1.zip › Appendix_A_Questionnaire.docx]

# Appendix A: Research Questionnaire

### I. Informed Consent Form

Dear Respondent,
Hello! Thank you for participating in this questionnaire survey regarding social media use and urban experiences in Dongguan.
This study aims to understand the exposure to Dongguan’s urban memory content on social media among migrants, as well as their feelings of place attachment and subjective well-being. The questionnaire is anonymous, and all data will be used solely for academic research. Participation is voluntary, and you may withdraw at any time.

**Q1. Have you read the above instructions and voluntarily agree to participate in this anonymous survey?**

A. I have read and agree to participate in this study.

B. I do not agree to participate in this study.
(Note: Participants who selected ‘A’ proceeded to the survey; those who selected ‘B’ were thanked and excluded.)

### II. Screening Questions

**Q2. What is your age?**

A. Under 18 years old (End of survey)

B. 18–25 | C. 26–30 | D. 31–40 | E. 41–50 | F. 51 and above

**Q3. Are you currently working, studying, or living in Dongguan?**

A. Yes | B. No (End of survey)

**Q4. What is your cumulative time working, studying, or living in Dongguan?**

A. Less than 6 months (End of survey)

B. 6 months to 1 year | C. 1–3 years | D. 3–5 years | E. 5–10 years | F. 10 years and above

**Q5. Are you a local resident born or raised long-term in Dongguan?**

A. Yes (End of survey)

B. No, I am a migrant who came to Dongguan from another place.

### III. Basic Demographic Information

**Q6. Gender:** A. Male | B. Female | C. Other

**Q7. Education:** A. Junior high or below | B. High school/Vocational | C. Associate degree | D. Bachelor’s | E. Master’s and above

**Q8. Marital Status:** A. Unmarried | B. Married | C. Divorced/Separated | D. Widowed

**Q9. Occupation:** A. Factory worker | B. Service industry | C. Self-employed | D. Administrative staff | E. Professional/Technical | F. Student | G. Unemployed | H. Other

**Q10. Household Registration (Hukou):** A. Dongguan Hukou | B. Non-Dongguan (Guangdong) | C. Out-of-province

**Q11. Living Arrangement:** A. Owned housing | B. Rented | C. Dormitory | D. Living with relatives/friends

**Q12. Monthly Income:** A. <3000 RMB | B. 3000–4999 | C. 5000–7999 | D. 8000–11999 | E. ≥12000

**Q13. Long-term Intentions:** (1 = No intention to 5 = Very clear intention)

### IV. Social Media Usage Characterization

**Q14. Frequency:** A. Never to E. Multiple times/day

**Q15. Duration:** A. <30 mins to E. >4 hours

**Q16. Platforms (Multiple Choice):** Douyin, Kuaishou, WeChat, Xiaohongshu, Weibo, Bilibili, Toutiao, etc.

**Q17. Encountered “Urban Memory” content in the past 3 months:** A. Often to D. Never.

**Q18. Formats:** A. Short videos | B. Graphic/Text | C. Live streams | D. Comments | E. Promo videos, etc.

**Q19. Content Interests:** A. History/Old areas | B. Industrial memory | C. Migrant workers’ stories | D. Intangible heritage, etc.

### V. Measurement Scales

Rating Scale: 1 = Strongly Disagree, 2 = Disagree, 3 = Neutral, 4 = Agree, 5 = Strongly Agree

| Code | Item Content |
| --- | --- |
| **SMU** | **Social Media Use Integration** |
| SMU1 | Social media has become a part of my daily life. |
| SMU2 | I often use social media to get local news or urban life information. |
| SMU3 | When I want to find information about Dongguan, I think of using social media. |
| SMU4 | I follow city topics related to my own life through social media. |
| SMU5 | Local content on social media has become part of my daily information sources. |
| **UMN** | **Perceived Urban Memory Narratives** |
| UMN1 | I have seen content about Dongguan’s history, culture, or urban changes on social media. |
| UMN2 | The urban memory content I am exposed to is usually presented in a story-based way. |
| UMN3 | The characters, locations, or scenes in this content feel authentic. |
| UMN4 | This content makes it easier for me to understand the past and present of Dongguan. |
| UMN5 | This content reminds me of my own life experiences in Dongguan. |
| UMN6 | I prefer urban memory narratives that feature real stories over simple city propaganda. |
| **NT** | **Narrative Transportation** |
| NT1 | When I watch or read content related to Dongguan’s urban memory, I am quite focused. |
| NT2 | I can imagine the Dongguan city scenes described in the content in my mind. |
| NT3 | Some content makes me feel as if I have entered the story’s situation. |
| NT4 | The characters’ experiences or urban changes in the content evoke my emotional reactions. |
| NT5 | After viewing such content, I sometimes continue thinking about the city of Dongguan. |
| NT6 | If the content is told vividly, I am willing to finish it or watch related content. |
| NT7 | This content makes me temporarily forget that I am just scrolling through my phone. |
| NT8 | This content makes me feel that Dongguan is not just a workplace, but a city with stories. |
| **PA** | **Place Attachment (Identity & Dependence)** |
| PA1 | I feel that Dongguan is a meaningful place to me. |
| PA2 | I feel a certain sense of closeness to Dongguan. |
| PA3 | Dongguan carries a significant part of my life experiences. |
| PA4 | When others talk about Dongguan, I sometimes feel this city is related to me. |
| PA5 | If I leave Dongguan in the future, I might miss certain places or experiences here. |
| PA6 | Dongguan holds a certain place in my personal experience. |
| PA7 | Dongguan can meet some of my current needs for work, life, or development. |
| PA8 | At the current stage, Dongguan is a suitable city for me to live or develop in. |
| PA9 | I have formed some familiar life circles or activity spaces in Dongguan. |
| PA10 | Compared to other cities, Dongguan better aligns with my current life needs. |
| PA11 | I have gradually adapted to the pace of life and environment in Dongguan. |
| PA12 | Dongguan provides living or development conditions that are hard to be replaced. |
| **UI** | **Urban Identity** |
| UI1 | I am willing to see myself as a member living in Dongguan. |
| UI2 | The identity of “a person living in Dongguan” has some meaning to me. |
| UI3 | When Dongguan receives positive evaluations from the outside, I feel happy. |
| UI4 | When Dongguan is misunderstood, I sometimes want to explain. |
| UI5 | I am willing to introduce Dongguan city stories to friends from out of town. |
| UI6 | I feel that my life experiences in Dongguan are part of my personal identity. |
| UI7 | I hope to gain a deeper understanding of the city of Dongguan. |
| **SWB** | **Subjective Well-being (Life Satisfaction)** |
| SWB1 | Overall, I am satisfied with my current life in Dongguan. |
| SWB2 | In most ways, my current life in Dongguan is close to my ideal. |
| SWB3 | I feel that my living status in Dongguan is generally acceptable. |
| SWB4 | So far, I have gained life experiences in Dongguan that are important to me. |
| SWB5 | If I could choose again, I would still consider coming to Dongguan to work or live. |
| **SS** | **Social Support** |
| SS1 | In Dongguan, I have someone I can talk to about life problems. |
| SS2 | When I encounter difficulties, there are people around who can offer help. |
| SS3 | I can get emotional support from friends, colleagues, or relatives in Dongguan. |
| SS4 | In Dongguan, I do not feel that I am always isolated and helpless. |
| SS5 | I have people I can trust in Dongguan. |
| SS6 | When I need advice, I can find someone to consult with in Dongguan. |

### VI. Attention Check Question

**Q27. To ensure questionnaire quality, please select “Agree” for this question.**
( ) Strongly Disagree | ( ) Disagree | ( ) Neutral | ( ) **Agree** | ( ) Strongly Agree
(Note: Respondents failing this check were excluded from the analysis.)

**Closing Remarks**
Thank you for your participation! Your responses are used solely for academic research and are presented in an aggregated format.
